# Supplementary material for: Monte Carlo dosimetric characterization of the IRAsource high dose rate Iridium-192 brachytherapy source: Comparison with the mHDR-v2r model
Source: PLoS One. 2026 Feb 6;21(2):e0339841. doi: 10.1371/journal.pone.0339841 (PMC12880691; doi:10.1371/journal.pone.0339841)
Supplement: Attachment — Submitted filename: Respons_to_Revewer.docx [file pone.0339841.s002.docx]

Response to Reviewer’s Comments

For Reviewer 1:

Reviewer #1: 1) The author performed Monte Carlo (MC) simulation close to the IRAsource at sub-millimeter spacial resolution. Please provide detailed description on how MC was implemented in those regions.

**Reply:**

Thank you for the helpful comments. In subsection EGS5 and EGS5MPI code, details about the EGS5 code (lines 37 to 53) have been provided. The energy deposit can be calculated by multiplying the stopping power, dE/dL, by the electron step length, dL. The distribution of electron step lengths, dL, is shown in Fig 3. The results show that, in the subsection “MC methods of dose rate,” a counting bin width of Δr = 0.0025 cm is sufficiently wide (lines 108 to 113).

2) The results showed that the absorbed dose (from both photon and electron sources) is lower than that from mHDR-v2r. In you Methods section, the author only simulated photons with energy greater than 10 keV. Please justify and explain why 10 keV cut-off energy was used. It is possible that the results of lower absorbed dose in this study is due to the exclusion of low energy photons (<10 keV).

**Reply:**

Thank you for your suggestion. Generally, a cut-off of 10 keV is appropriate for Monte Carlo simulation of brachytherapy dosimetry, as noted on page 31 of "Dose Calculation for Photon-Emitting Brachytherapy Sources with Average Energy Higher than 50 keV: Full Report of the AAPM and ESTRO

(<http://www.aapm.org/pubs/reports/RPT_229_full.pdf> )."

The cut-off energy was set at 10 keV by Sarabisl et al. [12] and Rostami et al. [13]. Hence, 10 keV was used. Incidentally, the cut-off energy was also set to 10 keV in the analysis of mHDR-v2r in the previous paper [3].

3) Line 119 to 122, the dose rate constant (1.112) from MCNP4C is between that (1.129) from HD-810 film and that (1.084) from EBT film, which contradicts to Line 122, "The value obtained from the MC simulation was higher". Please correct it.

**Reply:**

Thank you for your honest opinion. I wanted to show that it was the highest value among the MC simulations. To avoid any misunderstanding, I have rephrased it as follows:

According to previous studies, the dose rate constant reported by Sarabiasl et al. is (1.112 ± 0.005) cGyh^-1^U-^1^, whereas Rostami et al. reported a value of 1.1094 cGyh^-1^U^-1^. Ayoobian et al. reported a corresponding calculated value: (1.112 **±** 0.008) cGyh^-1^U^-1^ using MCNP4C. The value obtained from the MC simulation was higher than that of the other Monte Carlo (MC) simulations. Ayoobian et al. also reported two experimental results: (1.129 **±** 0.044) cGyh^-1^U^-1^ using HD-810 film, and (1.084 **±** 0.046) cGyh^-1^U^-1^ using EBT film. The results of this MC simulation were within the standard error margin of reported values.

4) Line 167 to 169, how would lack of actual IRA source measurement affects your results. The manuscript's result is purely from Monte Carlo without any experiment validation. The readers need to know how much error would be introduced to the results due to lack of accurate IRA source measurements.

**Reply:**

Thank you for your insightful comment. First, actual IRAsource measurement and Monte Carlo (MC) simulations should be completely independent. In other words, as long as the spectrum of photons (and electrons) from the source is determined, the absolute value of the absorbed dose can be calculated using MC simulation. In a previous study [3], the results of mHDR-v2r dosimetry was compared with those reported by Granero et al. and showed that the largest difference was approximately 2 %. Therefore, even with the IRAsource, the difference in results is expected to be within 2 %. This information has been included in the subsection Applicability of results (lines 207 to 210).

5)Line 199 to 200, In abstract, the author indicates that electron source is incorporated in MC simulation. However, in Line 199 to 200, it shows this incorporation will be future work. Please explain this conflicting information.

**Reply:**

Thank you for your honest opinion. My point is that more Monte Carlo (MC) simulations using different codes (e.g., other than MCNP and EGS5) and measurements for IRAsource are needed. For accurate MC simulation, incorporating source electrons into the MC simulation is desirable. This revision has been provided in lines 238 to 242.

For Reviewer 2:

Reviewer #2: Major points
• All mHDR-v2r data, methods, and figures are reused from their previous manuscript without citation (https://pubmed.ncbi.nlm.nih.gov/38335156/). Failure to disclose reused material may be considered self-plagiarism.

**Reply:**

Thank you for the advice. The following sentence has been included in the Result section:

The mHDR-v2r dosimetric values were taken from Reference [3] and compared with those of the IRAsource dosimetry.

Also, Fig 2 was designed from Fig 4 in Reference [3], and this revision has been indicated in the caption of Fig 2. In addition, references [3] are provided where the results are compared with those of mHDR-v2r.

• Include a side-by-side detailed figure containing both IRAsource and mHDR-v2r. If the source lengths are identical (3.5 mm) and the only structural difference lies in the end-cap thickness (0.53 mm for the IRAsource vs. 0.2 mm for the mHDR-v2r), and given that the IRAsource dosimetric protocol has already been published, the current study does not appear to offer substantial new information or novel insight.

**Reply:**

Thank you for the helpful comments. A schematic design of mHDR-v2r has been added to Fig 1. Since the capsule compositions of IRAsource and mHDR-v2r slightly differ, the composition ratio of mHDR-v2r has been added to the subsection “Description of source materials.” Regarding the comment "the current study does not appear to offer substantial new information or novel insight," The next response addresses this issue.

• The manuscript does not include any direct experimental measurement for the IRAsource. While comparisons to published data are performed, the absence of experimental benchmarks for the specific simulation configuration weakens the reliability of the conclusions.

**Reply:**

Thank you for the advice. As I have mentioned in the response to Reviewer 1, if source photons and electrons are prepared, the absolute value of the absorbed dose can be obtained using the Monte Carlo (MC) simulation alone. In other words, direct experimental measurements and MC simulation results should be completely independent. Conversely, normalizing the results of an MC simulation with actual measurements will reduce the reliability of the MC simulation. In a previous study [3], the results obtained were compared with those of Granero et al. for the mHDR-v2r source [2]. This approach was used to validate the results for the mHDR-v2r source. In this study, the same EGS5 code and main program were used as those in the previous study [3], except that the capsule geometry and capsule composition data from IRAsource were loaded during program execution instead of the mHDR-v2r source. This revision has been included in lines 196 to 202. The results of this study are reliable and valid because they were obtained in this manner. Sarabiasl et al. [12] and Rostami et al. [13] published the parameters for the IRAsource TG-43U1 protocol. However, the accuracy of their results, especially regarding the 2D anisotropy function, is questionable. Differences between the results obtained in the present study and those of Sarabiasl et al. or Rostami et al. provide substantial new information or novel insights.

• The study reports higher radial dose function values than other IRAsource MC studies, yet provides only a limited explanation for this discrepancy.

Reply:

Thank you for the insightful comments. I cannot explain why the results obtained in this study are higher than those of other IRAsource studies; nevertheless, the difference is minimal. The vertical scale makes the difference appear larger, whereas it is actually within 2%. This revision has been provided in lines 167 to 168. Additionally, the following sentence in the subsection “Applicability of results” has been deleted:

However, other results for IRAsource tended to show a larger discrepancy compared with mHDR-v2r.

• The anisotropy data shows deviations, particularly in the 0.25–1 cm range. The authors attribute this to capsule differences, but alternative explanations.

**Reply:**

Thank you for the opinion. As mentioned above, the results were obtained using the same program used to analyze the mHDR-v2r source [3]. The only difference is the geometry and composition of the capsules loaded during the calculation. Therefore, the difference in the 2D anisotropy function is mainly due to the difference in capsule geometry, or precisely, due to differences in capsule geometry and composition. However, considering that both capsules have the same density, 8.02 gcm^-3^ and are mainly composed of Fe, Cr, and Ni, the impact of differences owing to the capsule composition can be minimal. The following revision has been included in lines 193 and 194:

(To be precise, both the capsule geometry and composition contribute to the difference in results.)

Minor points
• Quantifiable results are lacking in the abstract section

**Reply**:

Thank you for the advice. In the abstract, the dose rate constant of the IRAsource obtained has now been provided. The difference in the 2D anisotropy function between the IRAsource and the mHDR-v2r source has also been provided in percentages.

• Line 19: The dimensions of 192IR “in IRAsource” are identical to those of mHDR-v2r.

**Reply**:

Thank you for the honest opinion. The sentence has been corrected by inserting "in IRA source."

• The conclusion refers to potential clinical utility, but without validation in a treatment planning system or comparison to measured data, this claim is premature.

**Reply**:

Thank you for your honest opinion. In the Conclusion section, the phrase "in the clinical application of IRAsource" has been revised to "in IRAsouce."

• Some references lack journal volume/issue/page numbers or are inconsistently formatted.

**Reply**:

Thank you for the advice. References [15], [16], and [17] do not follow the format because they are not commonly published articles in peer-reviewed journals. They can only be downloaded from the Internet, so I have included the URLs.
